# Typology of hyperthymic personalities with affective phases

**DOI:** 10.1192/j.eurpsy.2024.1362

**Published:** 2024-08-27

**Authors:** A. Barkhatova, A. Churkina, S. Sorokin

**Affiliations:** Department of endogenous mental disorders and affective states, Mental health research center, Moscow, Russian Federation

## Abstract

**Introduction:**

Modern authors characterize hyperthymic individuals as eloquent, humorous, self-confident, optimistic, energetic, liberated, sexually active, constantly planning and implementing their plans. Four or more of the listed characteristics indicate the individual’s involvement in the circle of hyperthymic people. Statistical data on the prevalence of hyperthymic is scarce, which is due to rare requests for help and the diagnosis of this condition not as a disease, but within the framework of characterological traits. Attempts to classify hyperthymics have been made more than once, but previously none of the authors divided them according to the presence of side character traits in the personality structure.

**Objectives:**

To establish psychopathological types of hyperthymic individuals in whom affective states were formed.

**Methods:**

The sample consisted of 50 patients (42 women, 8 men) who were on inpatient or outpatient treatment at the clinic since 2019 to 2022. Patients were examined by clinical-psychopathological, clinical-anamnestic methods due to the presence of a phase affective state.

**Results:**

Four types of hyperthymic personalities have been identified: anxious-hyperthymic, hysterical-hyperthymic, schizoid-hyperthymic and standard hyperthymic. *Anxious-hyperthymic type,* 20% (n=10) characterized by a combination of increased activity, sociability with such traits as suspiciousness, perfectionism, meticulousness, exactingness, concern for one’s health and the desire to maintain a healthy lifestyle. *Hysterical-hyperthymic type,* 46% (n=23) includes both hyperthymic and hysterical traits in the form of increased emotionality, egocentrism, drama, and desire for recognition from others. In addition, patients in this group are characterized by increased concern about their appearance (bright clothes, makeup, tattoos). *Schizoid-hyperthymic type,* 10% (n=5). In addition to increased activity and emancipation, patients in this group are prone to fantasizing, overvalued hobbies, sthenicity, emotional poverty and rationalism. *Standard type,* 24% (n=12) are characterized by the presence of typical hyperthymic traits - optimism, energy, constant desire for productive activity, success in the chosen profession, rapid career growth, sociability, openness.

**Conclusions:**

Hyperthymic individuals with the development of affective phases are heterogeneous in their psychopathological structure and have features of the pathocharacterological structure that make it possible to distinguish anxious-hyperthymic, hysterical-hyperthymic, schizoid-hyperthymic and standard types. The developed classification of hyperthymia reveals the predominance of the hysterical-hyperthymic type (46%).

**Disclosure of Interest:**

None Declared

